# The *Penicillium brasilianum* Histone Deacetylase Clr3 Regulates Secondary Metabolite Production and Tolerance to Oxidative Stress

**DOI:** 10.3390/jof8050514

**Published:** 2022-05-17

**Authors:** Daniel Yuri Akiyama, Marina Campos Rocha, Jonas Henrique Costa, Caroline Brandão Teles, Giuliana da Silva Zuccoli, Iran Malavazi, Taicia Pacheco Fill

**Affiliations:** 1 Department of Organic Chemistry, Institute of Chemistry, State University of Campinas, Campinas 13083-970, SP, Brazil; d195888@dac.unicamp.br (D.Y.A.); j161206@dac.unicamp.br (J.H.C.); 2 Department of Genetic and Evolution, Federal University of São Carlos, São Carlos 13565-905, SP, Brazil; marinacamposrocha@gmail.com; 3 Department of Biochemistry and Tissue Biology, Institute of Biology, State University of Campinas, Campinas 13083-970, SP, Brazil; teles.unicamp@gmail.com (C.B.T.); zuccoli.unicamp@gmail.com (G.d.S.Z.)

**Keywords:** epigenetic modulation, natural product biosynthesis regulation, reactive oxygen species

## Abstract

Most of the biosynthetic gene clusters (BGCs) found in microbes are silent under standard laboratory cultivation conditions due to the lack of expression triggering stimuli, representing a considerable drawback in drug discovery. To access the full biosynthetic potential, studies towards the activation of cryptic BGCs are essential. Histone acetylation status is an important regulator of chromatin structure, which impacts cell physiology and the expression of BGCs. In this study, *clr3*, a gene encoding a histone deacetylase in *Penicillium brasilianum* LaBioMMi 136, is deleted and associated phenotypic and metabolic changes are evaluated. The results indicate reduced growth under oxidative stress conditions in the ∆*clr3* strain, higher intracellular reactive oxygen species (ROS) levels, and a different transcriptional profile of 13 ROS-related genes of both strains under basal and ROS-induced conditions. Moreover, the production of 14 secondary metabolites, including austin-related meroterpenoids, brasiliamides, verruculogen, penicillic acid, and cyclodepsipeptides was evaluated in the ∆*clr3* strain, most of them being reduced. Accordingly, the addition of epigenetic modulators responsible for HDAC inhibition into *P. brasilianum*’s growth media also culminated in the reduction in secondary metabolite production. The results suggest that Clr3 plays an essential role in secondary metabolite biosynthesis in *P. brasilianum*, thus offering new strategies for the regulation of natural product synthesis by assessing chromatin modification.

## 1. Introduction

Filamentous fungi can produce a vast array of low molecular-weight molecules resulting from their secondary metabolism, aiding the fungus’ adaptation to environmental conditions, resisting and fighting back predators and competing microbes in their environmental niches [1]. These natural products possess a range of bioactive activities, ranging from the treatment of infectious diseases to potent toxic and carcinogenic properties [2]. Due to their important pharmaceutical potentialities, many efforts have been devoted in recent decades to identifying genes involved in the biosynthesis and regulation of these compounds [3].

*Penicillium brasilianum* presents a great biosynthetic ability. Secondary metabolites already reported as being produced by this species include diketopiperazines, polyketides, alkaloids, meroterpenoids, and cyclodepsipeptides [4]. Moreover, this fungus has demonstrated to be an important producer of potent convulsive and bacteriostatic brasiliamides [5,6,7,8], austin-related insecticidal meroterpenes [9,10,11], spirohexalines [10], and verruculogen-like tremorgenic alkaloids [9,12]. Functional analysis of *P. brasilianum*’s genome revealed 42 putative biosynthetic gene clusters (BGCs). Recently, Fill et al. reported the draft genome sequence of *P. brasilianum*, revealing a final assembly consisting of a genome size of ~32.9 Mbp [13]. AntiSMASH v3.0 analysis indicated the fungus genome presents 13 clusters related to the biosynthesis of potential polyketide compounds via PKS (polyketide synthase) enzymes; 12 different clusters involved in the production of secondary metabolites formed via NRPS-like (non-ribosomal peptide synthetases) enzymes, and 4 hybrid biosynthetic clusters, including the NRPS-terpene hybrid responsible for alkaloid biosynthesis, indicating the excellent, yet not fully explored, potential of this organism to produce bioactive secondary metabolites [13].

Bioinformatic, transcriptomic, and metabolomic analyses reveal that most microbial BGCs are not expressed when cultured in standard laboratory conditions due to the lack of abiotic and/or biotic stimuli present in their natural habitat, hindering our capability to fully access microbial biosynthetic potential [14]. Thus, novel strategies for the activation of BGCs are essential for natural product prospection.

Multiple factors regulate gene expression in a given condition, including chromatin packing. Histone modifications play an essential role in altering chromatin structure and, therefore, regulating transcription [15,16]. Histone acetylation is the most studied histone modification and depends on the concerted action of histone acetyltransferases (HATs) and histone deacetylases (HDACs) [17]. Histone hyperacetylation is known to induce gene-specific transcriptional activation in several organisms, thus being a solid strategy towards achieving structural diversity of natural products. Enhanced histone acetylation can be achieved by genetic approaches (such as gene deletion) or chemical inhibition of HDACs [18].

Based on the close relation between histone acetylation status and the expression of cryptic BGCs, the objective of this study is to evaluate the metabolic profile and phenotypic changes in a *P. brasilianum* ∆*clr3* mutant, which is a deletion strain of the class 2 histone deacetylase *hda1* homolog of *Saccharomyces cerevisiae*. Additionally, chemical epigenetic modulation was utilized as an alternative strategy for HDAC inhibition. Secondary metabolism changes were verified through different mass spectrometry-based approaches. Since HDACs regulate BGCs, these results indicate that genetic manipulation and pharmacological modification of chromatin acetylation are functional approaches to unveil secondary metabolite potential in *P. brasilianum,* allowing further studies in the prospection of novel natural products using this promising fungal model.

## 2. Materials and Methods

### 2.1. Fungal Strains and Culture Conditions

The fungus isolation procedure from the root bark of *Melia azedarach* was previously described [9]. *P. brasilianum* (LaBioMMi 136) [13] was cultivated on commercial potato-dextrose-agar (PDA) (Acumedia, San Bernardino, CA, USA) and potato-dextrose broth (PD) (Acumedia). Plates were inoculated with fresh conidia and grown at 30 °C for 7 days in darkness. Conidia were harvested by washing the agar surface with sterile distilled water and diluted to a final concentration of 10^5^ or 10^6^ conidia.mL^−1^.

### 2.2. Genomic DNA Extraction

The extraction of fungal genomic DNA was performed according to the method described by Malavazi and Goldman (2012) [19]. Briefly, conidia were incubated in 50 mL of PD at 25 °C, 150 rpm for 72 h. Mycelia were harvested, ground in liquid nitrogen, and suspended in 500 µL of Lysis buffer (200 mM Tris-HCl, 250 mM NaCl, 25 mM EDTA, 0.5% [*w*/*v*] SDS, pH 8.0). Genomic DNA purification was performed by phenol/chloroform extraction, followed by isopropanol precipitation. Purified DNA was air-dried and resuspended in ddH_2_O.

### 2.3. Construction of ∆clr3 Mutant

Construction of *the* ∆*clr3* mutant was performed following the protocol described by Malavazi and Goldman, 2012 [19]. The *clr3* deletion cassette used in this study was constructed by in vivo recombination in *S. cerevisiae* [19]. For the cassette construction, fragments of the 5′ and 3′ UTR regions that flanked the *clr3* gene were amplified from the genomic DNA of the wild-type strain. The primer sequences used in this study are listed in Table S1. Flanking regions contained a small sequence homologous to cloning sites of the pRS426 plasmid. The *hph* gene, which confers resistance to hygromycin, was PCR-amplified from the pAN7-1 plasmid and used as a selection marker in the deletion cassette. The three independent fragments, and *Bam*HI-*Eco*RI-cut pRS426, were transformed into the *S. cerevisiae* FGSC 9721 strain [19]. The plasmids containing the *clr3* deletion cassette were isolated using QIAprep Spin Miniprep Kit (QIAGEN, Hilden, NW, DEU) and used as templates to amplify the cassette using the outermost primers (5F and 3R). All PCR amplifications were performed using Phusion Flash High-Fidelity DNA Polymerase (Thermo Scientific, Waltham, MA, USA). The *clr3* deletion cassette was transformed into the *P. brasilianum* wild-type strain LaBioMMi 136 (Table S2) via protoplast transformation, as previously described [19]. Transformants were analyzed by diagnostic PCR and Southern Blot to confirm a single insertion at the *clr3* locus.

### 2.4. Southern Blot Analysis

Genomic DNA of the parental *P. brasilianum* and Δ*clr3* strains were isolated, as described above, and PstI-restricted. Chromosomal DNA fragments were separated on 1% agarose gel and blotted onto Hybond N^+^ nylon membranes (GE Healthcare, Chicago, IL, USA), following standard techniques [20,21]. Probe labeling for detection was performed using AlkPhos Direct Labeling and Detection System (GE Healthcare), according to the manufacturer’s description. A ChemiDoc™ MP imager (Bio-Rad, Hercules, CA, USA) was used for gel/blot documentation.

### 2.5. Phenotypic Assays for Oxidative Stress Sensibility

To monitor the growth of the Δ*clr3* and wild-type strains under oxidative stressing conditions, 1 × 10^4^ conidia of each strain were grown in 200 µL of PD in 96-well plates supplemented with varying concentrations of paraquat, menadione, H_2_O_2_, and the crop fungicides fludioxonil and iprodione (Figure 2) [22]. Plates were incubated for 72 h at 30 °C and photographed.

### 2.6. RNA Extraction and RT-qPCR Analysis

Samples subjected to oxidative stress caused by H_2_O_2_ were disrupted, and the total RNA obtained was extracted and processed for cDNA synthesis, as previously described [23,24]. The primers for the individual genes (Table S3) are listed in Table S4. At least three independent biological replicates were used for each condition and the relative fold change for each gene was calculated using comparative cycle threshold (*C_T_*), i.e., ΔΔ*C_T_*, analysis [25]. All the values were normalized to the expression of the *P. brasilianum tub1* gene, an ortholog of the *Aspergillus fumigatus tubA* gene [21]. Statistical analysis was performed by using one-way analysis of variance (ANOVA) with a Tukey’s post hoc test to assess differences in the mutant strain compared to the same condition in the wild-type strain.

### 2.7. In Silico Antioxidant Enzymes’ Subcellular Localization Prediction

Sub-cellular localization of protein products of all studied genes was predicted by DeepLoc-1.0 (http://www.cbs.dtu.dk/services/DeepLoc/), accessed on 13 March 2021.

### 2.8. ROS Quantification

Reactive oxygen species levels were quantified using the probe CM-H_2_DCFDA (Molecular probes, Invitrogen, Eugene, OR, USA). Both fungal strains were cultivated in minimum media for 24 h in a 96-well plate. Each test condition (different time of exposure to H_2_O_2_) was repeated in octuplicate. H_2_O_2_ was added to growth media to a final concentration of 5 mM before incubation with a 0.25 mM CM-H_2_DCFDA probe for 30 min at 30 °C, and then washed twice with Phosphate-Buffered Saline pH 7.4 (PBS). Fluorescence (485/535 nm) was measured immediately using Cytation^TM^ 5 Imaging Multi-mode Reader (BioTek, Winooski, VT, USA).

### 2.9. Secondary Metabolite Extraction

Cultivation was performed for both wild-type and Δ*clr3* strains, as previously described in Section 2.1. After incubation, the Petri dish content, including solid media and the fungal colony, was cut into small pieces and transferred into an Erlenmeyer flask. Extraction was performed using a solvent mixture consisting of methanol, ethyl acetate, and dichloromethane (1:2:3) [26]. Flasks were sonicated for 30 min in an ultrasonic bath and vacuum filtered. The extraction process was repeated twice. The solvent was removed under reduced pressure and the final extract was stored at −20 °C.

### 2.10. UPLC-DAD-MS Analyses

The chromatographic system was an ACQUITY^TM^ UPLC system (Waters, Milford, MA, USA) equipped with a diode array detection system. Waters Acquity UPLC BEH C18 analytical column (50 mm × 2.1 mm, 1.7 μm) was used as the stationary phase. The mobile phase was composed of 0.1% formic acid (A) and acetonitrile (B). Eluent profile (A/B %): 95/5 up to 2/98 within 8 min, maintaining 2/98 for 5 min and down to 95/5 within 1.2 min. Total run time was 18 min for each run and flow rate was 0.2 mL.min^−1^. The injection volume was 5 µL. Mass spectrometry detection was performed on a Xevo TQD mass spectrometer (Waters Corp., Milford, MA, USA) with an electrospray ionization (ESI) source. Analyses were performed in positive ion mode with *m*/*z* range of 100–1000; capillary voltage at 1.54 kV; and source temperature at 149 °C. MassLynx software (MassLynx, version 4.1, Waters, Milford, MA, USA) was used for data acquisition, equipment control, and spectra analysis.

### 2.11. High-Resolution Mass Spectrometry Analyses

Samples were diluted in methanol. High-resolution mass spectrometry analyses (HPLC-HRMS/MS) were performed in a Thermo Scientific QExactive© Hybrid Quadrupole-Orbitrap Mass Spectrometer. Analyses were performed in positive mode with *m*/*z* range of 133.4–2000; capillary voltage at 3.5 kV; source temperature at 250 °C; and S-lens 100 V. The stationary phase was a Thermo Scientific column Accucore C18 2.6 µm (2.1 mm × 100 mm × 1.7 µm). The mobile phase was 0.1% formic acid (A) and acetonitrile (B). Eluent profile (A/B %): 95/5 up to 2/98 within 10 min, maintaining 2/98 for 5 min, down to 95/5 within 1.2 min and maintaining for 8.8 min. Total run time was 25 min for each run and flow rate was 0.2 mL.min^−1^. Injection volume was 3 µL. MS/MS was performed by collision-induced dissociation (CID) with *m*/*z* range of 100–800 and the collision energy ranged from 10 to 50 V. MS and MS/MS data were processed with Xcalibur software (version 3.0.63) developed by Thermo Fisher Scientific, Waltham, MA, USA.

### 2.12. Chemical Epigenetic Modulation Experiments

Epigenetic modulation experiments were achieved by using suberoylanilide hydroxamic acid (SAHA) and nicotinamide (NAA) treatments alone, and combined using 48-well microplates. In each well, 1 × 10^5^ conidia were inoculated in 1 mL of PD medium containing 100 and 200 μM of NAA or SAHA, respectively. Cultures were incubated for 7 days at 30 °C (70 rpm). Extraction was conducted by liquid–liquid partition after transferring the content of each well to separation funnels using ethyl acetate (3 times of 2 mL). The organic phase was dried under reduced pressure, and the extracts were analyzed by HPLC-HRMS/MS.

### 2.13. Mass Spectrometry Imaging (MSI)

MSI analyses were performed directly on the agar surface using a Prosolia DESI source Modelo Omni Spray 2D^®^-3201 coupled to a Thermo Scientific QExactive^®^ Hybrid Quadrupole-Orbitrap Mass Spectrometer. DESI configuration used was the same set by Angolini et al. [27]. The methanol flow rate was set at 10.0 mL min^−1^. MS data were processed with Xcalibur software (version 3.0.63) developed by Thermo Fisher Scientific. IMS data was acquired using a mass resolving power of 70,000 at *m*/*z* 200. DESI-MSI data was converted into image files using Firefly data conversion software with a bin width of ∆*m*/*z* ± 0.03 (version 2.1.05) and viewed using BioMap software (version 3.8.0.4) developed by Novartis Institutes, Cambridge, MA, USA for Bio Medical Research. Color scaling was adjusted to a fixed value for the comparison between the samples.

## 3. Results and Discussion

### 3.1. Δclr3 Strain Construction and Phenotypic Analysis

To better access the *P. brasilianum* cryptic natural products, activation of silent BGCs is one of the approaches to dissect the identity of natural products of this organism. Modification of chromatin landscape has been a widely used strategy to achieve metabolic diversity in fungi [28]. Histone deacetylase activity inhibition, either through gene deletion or epigenetic modulation, has presented relevant results in altering fungal secondary metabolism [29,30] and, in some phytopathogenic species, such as *Fusarium fujikuroi*, virulence properties have been altered [31]. Similarly, the deletion of the *hdaA* gene in *P. chrysogenum* resulted in significant expression changes of genes related to pigment production and the upregulation of a sorbicillinoids BGC [32], indicating that HDAC inhibition is a feasible strategy in the *Penicillium* genera.

Based on the genome annotation in the NCBI database, four HDACs were identified in the *P. brasilianum* genome. To further identify and analyze each HDAC, Blastp searches and phylogenetic analyses with the amino acid sequences of known HDACs from other fungal species were performed. A phylogenetic tree was constructed using MEGA6 software based on the alignment of amino acid sequences (Figure S1). Altogether, the following HDAC homologue genes were identified: *hosB* (PEBR_24088); *sir2* (PEBR_32801); *clr3* (PEBR_10023); and *rpdA/rpd3* (PEBR_38155).

Classical HDACs can be grouped into four classes (I, II, III, and IV) based on the homology of its catalytic domain to yeast HDACs [33]. The gene *P. brasilianum* PEBR_10023, termed here as *clr3*, putatively encodes an ortholog of the class II *had1* histone deacetylase gene in *S. cerevisiae* revealing 40% identity and 59% similarity (3.10^−170^ *e*-value), as shown in Figure S1.

Further sequence analyses also indicated that the *clr3* gene presented significant sequence identity and similarity with other known HDACs previously characterized in other filamentous fungi, such as the *hdaA* in *A. fumigatus* (68% identity, 60% similarity, 0 *e*-value); *A. nidulans* (65% identity, 78% similarity, 0 *e*-value); and *P. chrysogenum* (69% identity, 81% similarity, 0 *e*-value) [32,34,35]. The *hdaA* knockout strains for these species presented different fungal development and metabolic profiles. In *P. chrysogenum*, *hdaA* deletion resulted in the transcriptional activation of sorbicillinoids biosynthesis [32]. The *A. nidulans* Δ*hdaA* strain presented higher sensitivity to oxidative stressing conditions [34], while the null mutant in *A. fumigatus* displayed a decreased germination rate and reduced vegetative growth. In addition, *A. fumigatus hdaA* negatively regulates the expression of four NRPS clusters [35].

**Figure 1:**
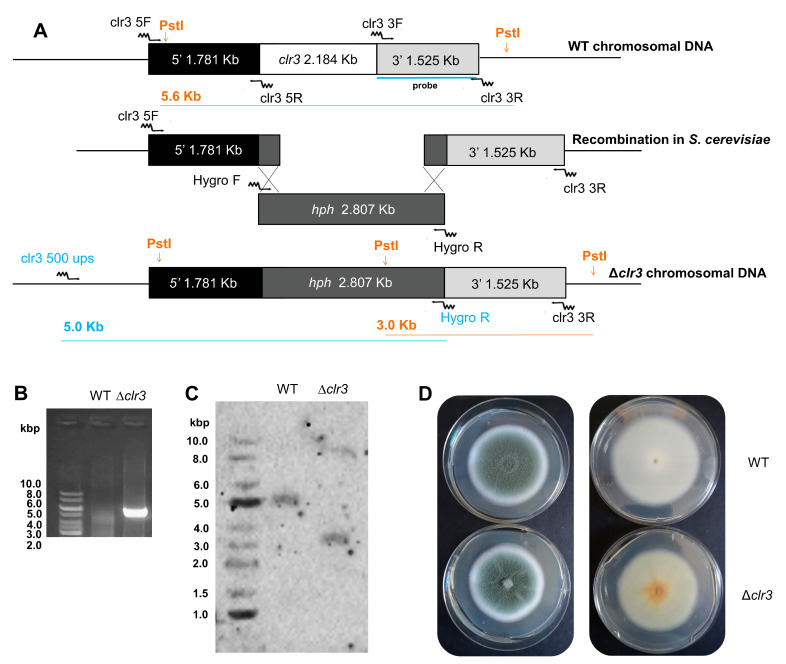
Construction of *clr3* deletion mutant. Gene replacement strategy for *clr3* deletion, in which the *hph* gene was used as a selection marker. The primer names and annealing regions are indicated by arrows (primer sequences are described in Table S1). Deletion cassettes were constructed by in vivo recombination in *S. cerevisiae* (**A**). Diagnostic PCR was performed to evaluate *clr3* loci after gene replacement using primers located 500 bp upstream of the deletion cassette, shown in blue letters and arrows. No amplification was observed in the wild-type (WT) strain (**B**). Southern blot analysis indicating the hybridization of the probe, which recognizes the region indicated by orange letters and lines (**C**). Phenotype of the Δ*clr3* and wild-type (WT) strains grown in PDA at 30 °C for 7 days (**D**), top (left) and bottom (right) of the colony.

To evaluate the impact of HDAC activity in the biology of *P. brasilianum* and in the secondary metabolism profile, a *clr3* deletion strain was constructed. Gene deletion was achieved through homologous recombination using a deletion cassette constructed in vivo in *S. cerevisiae* harboring the *hph* gene (hygromycin B phosphotransferase), as a selection marker. Gene deletion strategy and diagnostic PCR are shown in Figure 1A,B. Southern Blot analysis confirmed the *clr3* deletion via a single copy integration of the deletion cassette at the *clr3* locus (Figure 1). One of the mutant strains was chosen and further used for phenotypic and the analyses of secondary metabolites.

**Figure 2:**
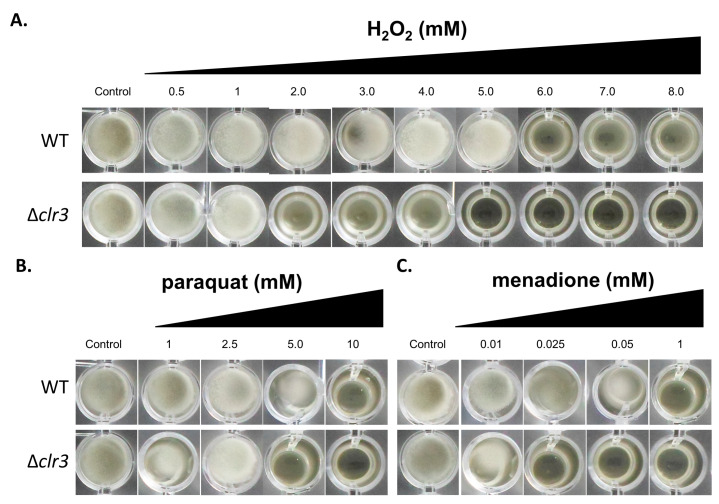
*clr3*-null mutants exhibited sensibility to oxidative stress caused by H_2_O_2_, paraquat, and menadione. The 1 × 10^4^ conidia of wild-type (WT) and mutant strains were inoculated in 200 µL of PD broth (96-well plates) supplemented or not with varying concentrations of (**A**) H_2_O_2_, (**B**) paraquat, and (**C**) menadione. Plates were incubated at 30 °C for 72 h and then photographed.

After *clr3* knockout confirmation, phenotypic assays were performed to evaluate possible changes in fungal physiology in the mutant strain. Our phenotypic analyses revealed that the Δ*clr3* mutant is more sensitive to the oxidative damaging compounds tested, such as H_2_O_2_, paraquat, and menadione (Figure 2). Interestingly, higher levels of susceptibility were observed in the mutant strain exposed to H_2_O_2_.

Oxidative stress results from an imbalance between pro-oxidant species and the levels of antioxidant defenses, resulting from the generation of reactive oxygen species (ROS). In contrast to *Aspergillus* ssp., minimal data are available on the antioxidative defense system of *P. brasilianum*. For instance, in *A. nidulans*, expression analysis revealed that catalase *catB* is upregulated upon ROS increase in the wild-type strain, but not in the Δ*hdaA* mutant [34], suggesting that chromatin modification is part of the regulatory mechanism against oxidative stress. As CatB is one of the known enzymes responsible for detoxifying hydroperoxides in *A. nidulans* hyphae, the authors hypothesize that a positive failure in the positive expression of CatB is one of the main reasons for the sensitivity of Δ*hdaA* strains against ROS in this fungus [36]. Our data suggest that a similar scenario occurs upon the deletion of *clr3* in *P. brasilianum*, since peroxidases may be the most affected ROS detoxifying enzymes in the Δ*clr3* mutant, considering the results obtained for the H_2_O_2_ challenge. In summary, our phenotypic analysis suggested that *clr3* was relevant in both conditions where the highly membrane-diffusible H_2_O_2_ molecule was exogenously added to the cells and when cells were challenged with paraquat and menadione, two compounds that continuously generated superoxide and H_2_O_2_ by the redox cycling in the mitochondria [37].

In both *Trichoderma atroviride* and *A. nidulans*, the deletion of histone deacetylases caused reduced growth under oxidative stressing conditions compared to their respective parental strains [32,35,38]. The mechanism underlying oxidative stress response is particularly relevant in phytopathogenic fungi, since most hosts’ responses to fungal infection are based on ROS production by the plant and counteracting responses from the fungus [38]. Different *P. brasilianum* strains have already been reported as onion (*Allium cepa* L.) pathogens [39], but little is known about this host–pathogen interaction and the virulence attributes of this fungus, which may involve the function of *clr3*.

### 3.2. The Transcriptional Basis for P. brasilianum Δclr3 Sensitivity to Hydrogen Peroxide

**Figure 3:**
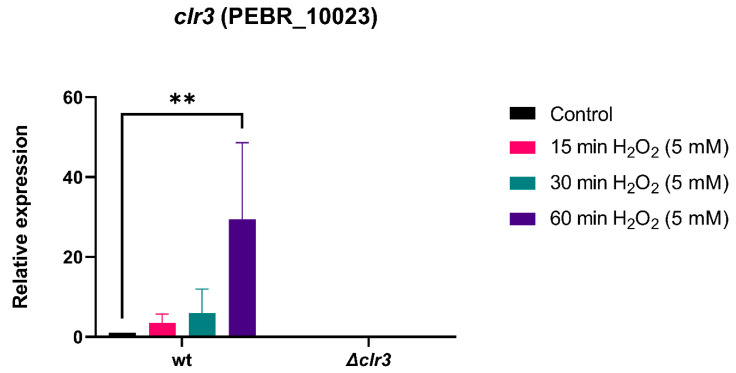
*clr3* is induced during oxidative stress in the wild-type (wt) strain. Expression of *clr3* was investigated by RT-qPCR in the strains subjected to oxidative stress caused by hydrogen peroxide during the indicated time points at 30 °C. Values represent the average of the results from three independent experiments with two technical repetitions The error bars represent standard deviation, ** *p* ≤ 0.01 (one-way ANOVA, significance level 0.05).

The *P. brasilianum* Δ*clr3* strain was shown to be more sensitive to H_2_O_2_ (Figure 2A). Therefore, an RT-qPCR approach was used to characterize the transcriptional basis of the oxidative stress sensitivity of the Δ*clr3* mutant (Figure 3).

About 30-fold increase in *clr3* expression was observed in the wild-type strain after 60 min of exposure to 5 mM H_2_O_2_, indicating that *clr3* plays an important role in positively regulating the antioxidant defense system of *P. brasilianum* against H_2_O_2_. Similar results were observed in *T. atroviride*, in which the expression of the histone deacetylase-encoding gene *hda-2* was also increased under the influence of H_2_O_2_ and menadione [40].

Several homolog genes identified in *A. fumigatus* as being involved in oxidative stress tolerance were identified by Blastp searches in the *P. brasilianum* genome (Table S4), and sequences were used to design primers for the detection of mRNA abundance in response to H_2_O_2_ (5 mM) in the wild-type and Δ*clr3* mutant.

Peroxiredoxins (Prxs) are cysteine-based peroxidases containing one (1-Cys) or two (2-Cys) catalytic cysteine residues that contribute for maintenance of intracellular peroxide levels and hydroperoxide removal under various subcellular locations [41]. The *P. brasilianum* genome possesses two 1-Cys Prxs, PEBR_28450 and PEBR_20685; three genes encoding putative enzymes from the PRX5-subfamily, PEBR_37093, PEBR_01141, and PEBR_39037; as well as a glutathione peroxidase: PEBR_14961. We named these genes *prx1*, *prx2*, *prxA*, *prxB*, *prxC*, and *hyr1*, respectively. Protein products for the orthologs of *prx1* and *prxA* in *A. fumigatus* (Table S4) have been reported as being differently synthesized after fungal incubation with 2 mM of H_2_O_2_, indicating an associated role in oxidative stress response [42].

Superoxide dismutase (Sods) play an essential role in the detoxification of highly reactive superoxide anions [43]. Sods representatives in the *P. brasilianum*’s genome, including one copper-zinc (PEBR_25708), one manganese (PEBR_04850), and one iron (PEBR_29521) Sod, were identified and named *sod1*, *sod2*, and *sod3*, respectively. The deletion of orthologs for these genes in *A. fumigatus* led to high sensibility to superoxide ions produced by menadione [43]. Lastly, our searches for catalases identified four genes, PEBR_36641; PEBR_24619; PEBR_24196; and PEBR_36037, named as *cta1, cta2, catA*, and *catB,* respectively. Deletion mutants for the respective orthologs of these genes in *A. fumigatus* also presented higher sensitivity to H_2_O_2_ [44].

**Figure 4:**
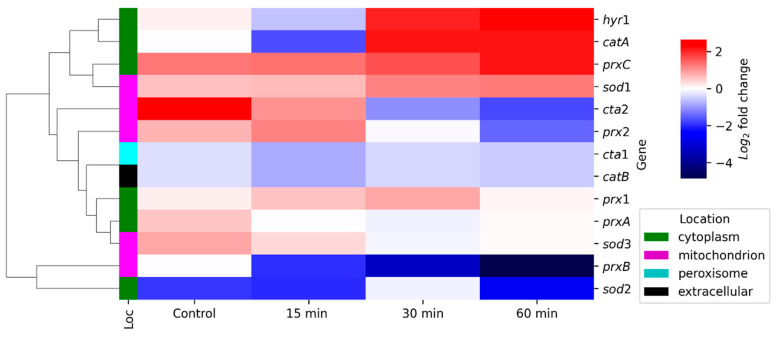
*clr3* deletion affects mRNA accumulation of genes encoding ROS detoxifying enzymes including catalases, superoxide dismutases and peroxiredoxins. Expression of oxidative stress genes were investigated by RT-qPCR in the strains subjected to oxidative stress induced by H_2_O_2_ (5 mM) during the indicated time points (minutes) at 30 °C. Hierarchical clustering data represent the log 2-fold changes in gene expression in the ∆*clr3* mutant compared to the wild-type strain. Values represent the average value of three independent experiments with two technical repetitions each (see Figure S2). In silico prediction of subcellular location for each enzyme is also shown on the left of the heat map.

The expression analyses of the above-selected oxidative stress response genes indicate a broad range of results in response to H_2_O_2_ treatment (Figure 4). Both up and downregulated genes were identified across different enzyme families, through different time points. To better address our findings, in silico predictors of protein subcellular localization were utilized to look for patterns between enzyme localization and mRNA abundance (Table S3).

In silico predictions indicated that the ROS detoxifying enzymes analyzed here reside on cytoplasm, mitochondrion, peroxisome, and one of them secreted to the extracellular environment. However, the hierarchical clustering did not return a clear correlation between the mRNA levels of each enzyme and their subcellular localization, thus indicating a complex antioxidant regulation system and a potential regulatory role of *clr3* upon different classes of antioxidant enzymes. Both enzymes, encoded by *catB* and *cta1*, respectively, were downregulated in the Δ*clr3* strain across all time points, as well as *prxB* and *sod2*. Based on our findings, we hypothesize that *clr3* is an important regulator for these genes. Whether the control exerted by *clr3* on these genes is direct or indirect remains to be determined.

Interestingly, *clr3* negatively controls the expression of some other antioxidants, such as the cytoplasmic *hyr1*, *catA*, *prxC*, and mitochondrial *sod1*, which presented increased mRNA abundance in the deletion strain, suggesting that the lack of Clr3 activity triggers a compensatory effect to maintain ROS homeostasis, especially in cytosol. In the mitochondria, *cta2* and *prx2* activity was upregulated during the first 15 min of cell exposure to H_2_O_2_ and downregulated in later stages of exposure, implying their important role in the early stages of ROS accumulation. For the later time points, cytoplasmic glutathione peroxidase *hyr1* and *catA* mRNA levels were increased in the null mutant after 30 min of H_2_O_2_ incubation.

Interestingly, *prxC*, *sod1*, *cta2*, *prx2*, *cta1*, *catB*, *prx1*, *prxA*, *sod3*, and *sod2* presented different expression levels in the mutant strain, even under non stressing conditions. Tribus and colleagues addressed a similar finding in their evaluation on the effect of HdaA deletion in *A. nidulans* growth under oxidative stress [34]. In their study, Δ*hdaA* strain grown in the absence of ROS-generating drugs presented higher levels of protein carbonylation, which is a measure of the overall oxidative damage within the cells [45].

**Figure 5:**
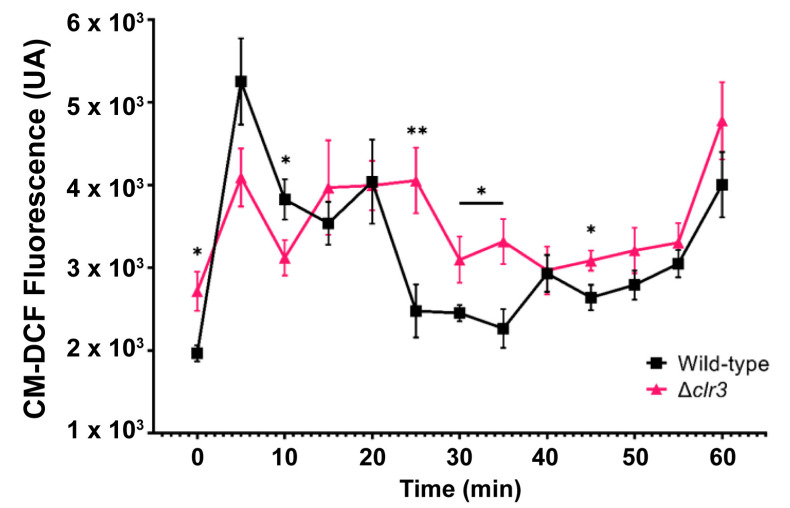
Intracellular ROS levels in the wild-type and mutant strains without (0 min) and after exposure to 5 mM of H_2_O_2_. The data represent the average value of eight biological replicates. The error bars represent the standard deviation, * *p* ≤ 0.05, ** *p* ≤ 0.01 (one-way ANOVA, significance level 0.05). Both fungal strains were cultivated in minimum media for 24 h in a 96-well plate. H_2_O_2_ was added to growth media to a final concentration of 5 mM before incubation with 0.25 mM CM-H_2_DCFDA probe for 30 min at 30 °C and then washed twice with PBS.

To evaluate the intracellular levels of ROS in the *P. brasilianum* Δ*clr3* strain, we fluorometrically monitored the ROS formation in the wild-type and mutant strains through the oxidative conversion of non-fluorescent CM-H_2_DCFDA into fluorescent CM-DCF (Figure 5).

As predicted in our qPCR analyses, intracellular ROS levels in the absence of H_2_O_2_ were higher in the null mutant than in the wild-type strain. Consistently, the expressions of *sod2*, *cta1*, and *catB* were significantly downregulated under basal conditions. The overall increase in the transcription of *hyr1, catA, prxC*, and *sod1* after 30 min (Figure 4) was not sufficient to lower the ROS levels in the Δ*clr3* strain, again suggesting multiple regulatory mechanisms, which are possibly deficient in the mutant.

### 3.3. Natural Product Diversity in Δclr3 Strain

To further probe the contribution of *clr3* in *P. brasilianum* physiology, we subsequently used the Δ*clr3* mutant to investigate the secondary metabolite production as an initial approach to natural product discovery in this species. For metabolic profile comparison, wild-type and Δ*clr3* strains were grown in identical cultivation conditions and crude extracts were analyzed by UPLC-DAD. The resulting chromatograms (Figure S3) were plotted to the same scale for a better comparison.

We observed no alteration in the chromatogram profile between the two strains, indicating that the deletion of *clr3* did not induce the production of new metabolites or repression of those constitutively formed in our culture conditions. On the other hand, peak areas were significantly different for both strains, indicating that *clr3* has an important regulatory role in secondary metabolite production.

**Figure 6:**
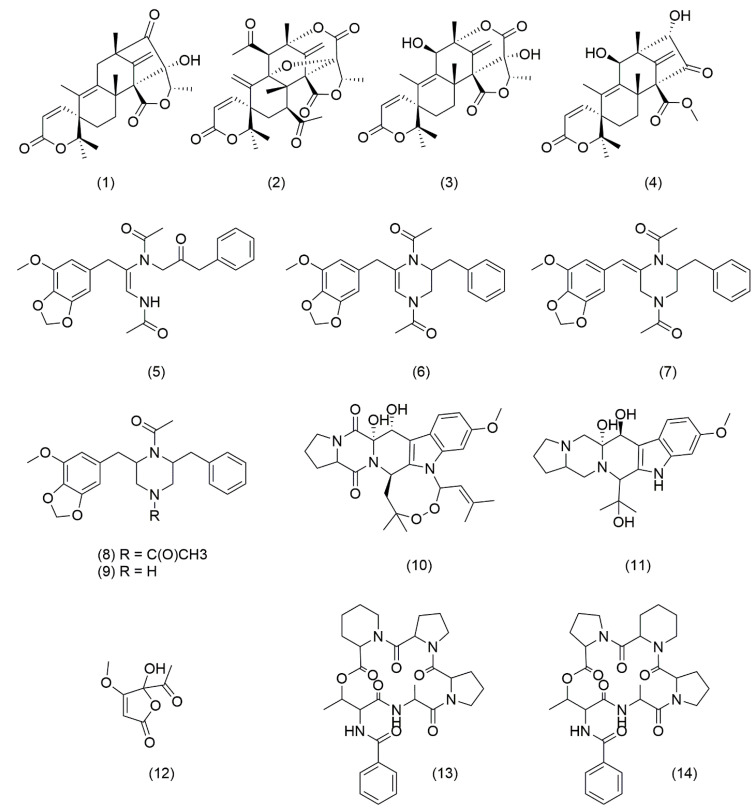
Chemical structures of metabolites annotated in this study.

To identify the molecules with different production levels in both strains, crude extracts were analyzed further by HPLC-HRMS/MS, and natural product dereplication was performed by manually searching on Natural Products databases. Furthermore, the obtained data were compared to HRMS data from previous related studies on *P. brasilianum* secondary metabolism [6,7,8,9,10,11,12,32,46,47,48,49]. Interestingly, a total of 15 differentially produced compounds already known to be produced by *P. brasilianum* were putatively identified. HRESI-MS data for all metabolites described can be found in Table 1, Figure 6, and Figures S4–S17.

Austin-related meroterpenoids numbered (Table 1) have previously been related to both *Aspergillus* and *Penicillium* genera, presenting various biological activities, such as convulsive and insecticidal [8,9,36,46]. Brasiliamides are rare examples of fungal phenylpropanoids produced by *P. brasilianum*, possessing convulsive and a weak antibacterial activity [7,8]. Verruculogen is a common tremorgenic mycotoxin also produced by both the *Aspergillus* and *Penicillium* genera [9,47]. Furthermore, verruculogen derivatives, such as verruculogen TR-2, have also had their production induced in *P. brasilianum* [12]. Penicillic acid is a polyketide produced by many strains of *Penicillium* fungi, being an important mycotoxin with antibacterial activity [50]. Cyclodepsipeptides JBIR 114 and JBIR 115 present a unique structure with three neighboring cyclic amino acids, one proline and two pipecolinic acids, indicating a great NRPS-like enzymatic potential in *P. brasilianum* [51].

The lack of novel-induced secondary metabolites due to *clr3* deletion was unexpected based on its transcription role to suppress gene expression and may be highly related to our cultivation condition, again suggesting the complexity of secondary metabolite production. Nonetheless, there are several reports in the literature demonstrating that the perturbation of HDACs activity has led to both the up- and downregulation of a number of BGCs, the suppression of a metabolite, or the induction of new molecules, thus indicating a complex response mechanism to chromatin landscape modification in natural product biosynthesis [50,51,52].

Since the deletion of *clr3* did not induce the production of any new metabolites, different approaches to modulate chromatin structure were sought. In addition to gene deletion, HDAC inhibition can be achieved through chemical epigenetic modulation [28,53]. In *Aspergillus niger*, growth in media supplemented by suberoylanilide hydroxamic acid (SAHA), a HDAC inhibitor, was able to alter its secondary metabolism and induce a new pyridine [54]. In the *Penicillium* genera, the same strategy was applied in *Penicillium mallochii*, resulting in two new natural sclerotioramine derivatives [55].

In this study, two chemical epigenetic modulators were used, NAA and SAHA, as well as a mixture of both. Extracts from the fungus grown in the presence of these epigenetic modulators were analyzed through HPLC-HRMS/MS. Previously dereplicated compounds were also detected in these experiments, although production levels varied through the different modulators.

### 3.4. The Regulatory Role of HDACs in Penicillium brasilianum Secondary Metabolism

**Figure 7:**
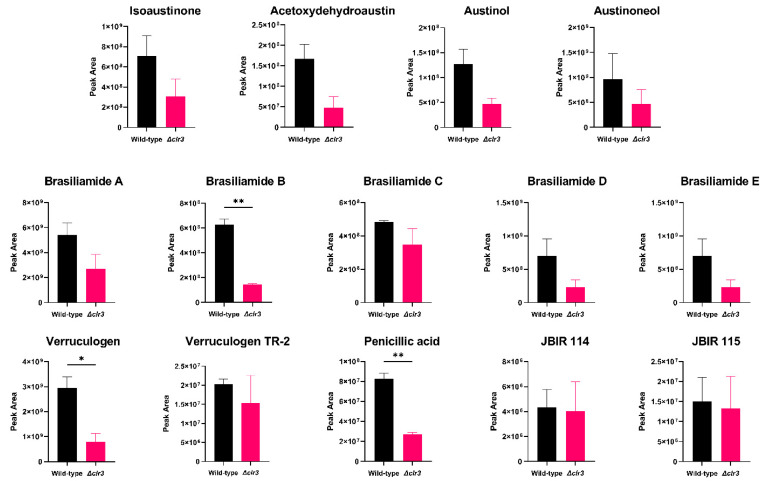
Relative quantification of metabolites in both wild-type and Δ*clr3* strains. The data represent the average value of two replicates. The error bars represent the standard deviation, * *p* ≤ 0.05, ** *p* ≤ 0.01 (unpaired *t*-test, significance level 0.05).

**Figure 8:**
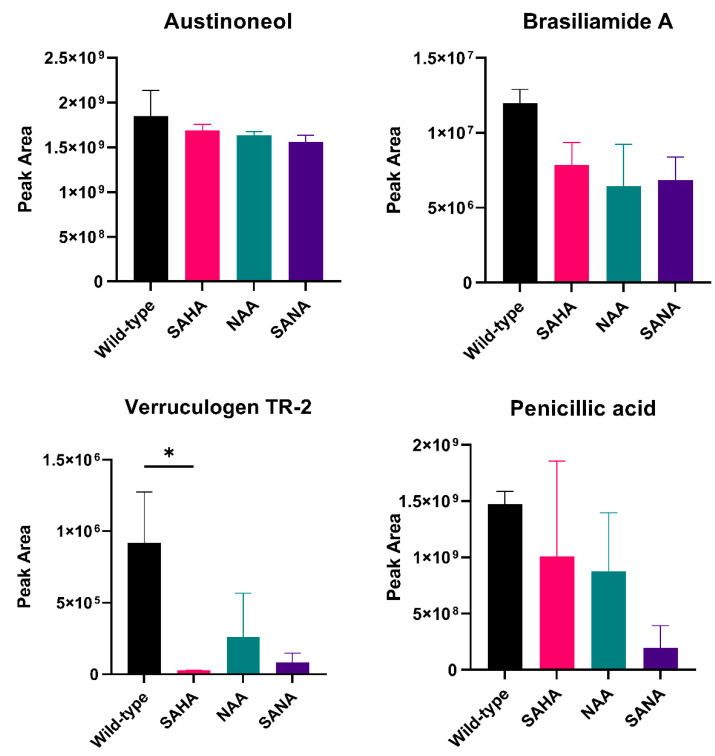
Relative quantification of detected metabolites in extracts from *P. brasilianum* grown in the presence of suberoylanilide hydroxamic acid (SAHA), nicotinamide (NAA), as well as a mixture of both (SANA). The data represent the average value of two replicates. The error bars represent standard deviation, * *p* ≤ 0.05 (one-way ANOVA—Tukey’s test, significance level 0.05).

To better understand the magnitude of HDAC regulation of the secondary metabolism in *P. brasilianum*, and to compare HDAC inhibition through *clr3* deletion and chemical epigenetic modulation, metabolite quantification was performed. Relative quantification of the metabolites was conducted by comparing integrated peak areas of the identified metabolites in the HPLC-HRMS/MS analyses of the crude extracts from both strains (Figure 7), as well as the ones obtained from the chemical epigenetic modulation experiment (Figure 8).

By comparing the secondary metabolism of both wild-type and Δ*clr3* strains*,* significant production level differences can be noted. Notably, brasiliamide A was the most produced amide in both extracts; however, its biological role has not yet been fully unveiled. This is the first report in which brasiliamide production has been evaluated upon HDAC-inhibition conditions.

Similarly, the deletion of SntB, a global histone deacetylase inhibitor in *A. flavus*, resulted in the downregulation of the flavotoxin BGC [56]. In our study, HDAC inhibition also caused the downregulation of mycotoxins, such as verruculogen and penicillic acid. On the other hand, isoaustinone, brasiliamide D, verruculogen TR-2, JBIR 114, and JBIR 115 presented remarkably similar production levels, indicating a low correlation between Clr3 activity and the biosynthesis of these natural products.

In *A. fumigatus,* wild-type, Δ*hdaA* mutants and the over-expression of Δ*hdaA*-complement strains exhibited significant differences in their secondary metabolism profiles, which also resulted in altered virulence properties [35]. Ethyl acetate extracts from each strain were added to macrophages and the *hdaA* over-expression strain induced cell death at equivalent biomass [35]. Albeit the metabolism of the *P. brasilianum* Δ*clr3* strain was not evaluated in vivo, the metabolic changes observed here might alter its endophytic and pathogenic properties. Further studies are necessary to evaluate this proposition.

Regarding the chemical HDAC modulators, the most significant differences were observed in the verruculogen TR-2 concentration, in which the mixture of modulators caused a total suppression of its production. Penicillic acid and brasiliamide A also presented significant differences, especially under the combination of SAHA and NAA.

**Figure 9:**
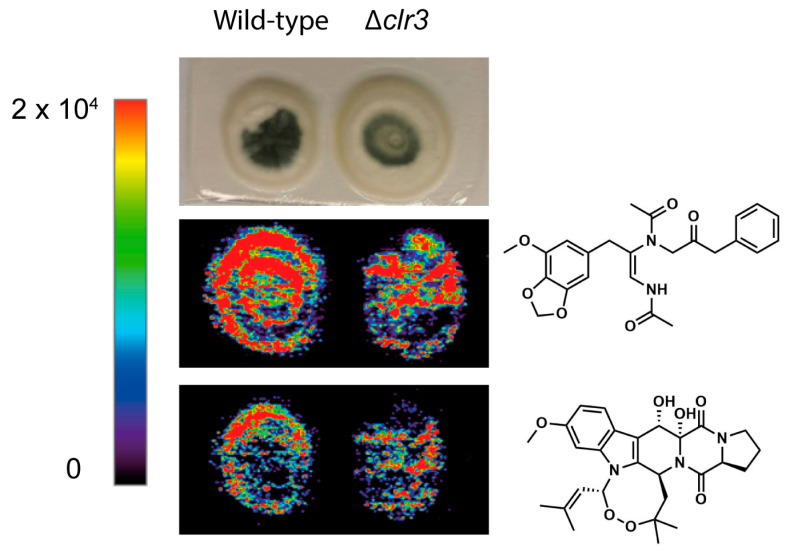
(+) DESI-MSI showing different spatial distributions and concentrations of brasiliamide A and verruculogen on fungal surface. Images are plotted on the same color scale from 0 (black) to 2 × 10^4^ (red); ion concentration cannot be compared across images due to ionization differences between molecules.

As an alternative approach to validate these findings, Mass Spectrometry Imaging (MSI) was used to confirm the metabolic production differences of brasiliamide A and verruculogen (Figures S18 and S19), the two most produced metabolites in *P. brasilianum* in this study (Figure 9), as well as to monitor the spatial distribution of both molecules in the fungal colony.

DESI-MSI analyses of wild-type and Δ*clr3* strains indicated a lower accumulation of brasiliamide A and verruculogen in the colony surface of Δ*clr3* than in the wild type. Additionally, the DESI-MSI images indicate that the production of these secondary metabolites occurs in both the conidia and hyphal tissue since the detection occurred throughout the colony, including areas of lower conidiation.

Based on the metabolomic approaches, it was possible to verify a close relation between Clr3 activity and secondary metabolite production in *P. brasilianum*. Both the deletion of *clr3* and chemical epigenetic manipulation led to a downregulation of secondary metabolite production, indicating that histone deacetylases play an important role in regulating the *P. brasilianum* secondary metabolism regulation.

## 4. Conclusions

Understanding filamentous fungi secondary metabolism and its regulation by chromatin structure is an important step towards natural product discovery. Here, we demonstrated for the first time the effect of HDAC inhibition on *P. brasilianum’s* development and secondary metabolite production. In terms of fungal development, the Δ*clr3* strain exhibited sensitivity in growth under oxidative stress conditions, higher ROS levels in basal and ROS-induced conditions, presenting different transcript levels for *clr3* and crucial ROS-related genes compared to the parental strain. Few genes were also upregulated in the null mutant, likely compensating for the lack of *clr3* activity. Based on the metabolic approaches, both the deletion of *clr3* and chemical inhibition of HDACs caused the reduction in the production of secondary metabolites, such as austin-related meroterpenoids, brasiliamides, verruculogen, penicillic acid, and cyclodepsipeptides. Finally, the paradoxical relationship between higher histone acetylation status and decrease in natural product biosynthesis might be related to the fungal requirements to direct cell energy sources to ROS detoxification, rather than eliciting secondary metabolite pathways, at least in in vitro conditions [57].

## Figures and Tables

**Table 1:** HRESI-MS data obtained for all annotated compounds.

N°	Molecule	Ion Formula ([M+H]^+^)	Calculated *m*/*z* ([M+H]^+^)	Experimental *m*/*z* ([M+H]^+^)	Error (ppm)	Class
**1**	Isoaustinone	C_25_H_31_O_6_	427.2115	427.2116	0.15	Meroterpenoid
**2**	Acetoxydehydroaustin	C_29_H_33_O_11_	557.2017	557.2017	−0.03	Meroterpenoid
**3**	Austinol	C_25_H_31_O_7_	443.2064	443.2064	−0.09	Meroterpenoid
**4**	Austinoneol	C_24_H_31_O_6_	415.2115	415.2115	0.08	Meroterpenoid
**5**	Brasiliamide A	C_24_H_27_N_2_O_6_	439.1864	439.1865	−0.05	Bisphenylpropanoid amides
**6**	Brasiliamide B	C_24_H_27_N_2_O5	423.1914	423.1914	−0.09	Bisphenylpropanoid amides
**7**	Brasiliamide C	C_24_H_27_N_2_O_5_	423.1914	423.1915	0.06	Bisphenylpropanoid amides
**8**	Brasiliamide D	C_24_H_29_N_2_O_5_	425.2071	425.2070	−0.16	Bisphenylpropanoid amides
**9**	Brasiliamide E	C_22_H_27_N_2_O_4_	383.1965	383.1966	0.24	Bisphenylpropanoid amides
**10**	Verruculogen *	C_27_H_32_N_3_O_6_	494.2286	494.2287	0.28	Diketopiperazines
**11**	Verruculogen TR-2	C_22_H_28_N_3_O_6_	430.1973	430.1969	−0.84	Diketopiperazines
**12**	Penicillic acid	C_8_H_11_O_4_	171.0652	171.0652	0.20	Polyketide
**13**	JBIR 114	C_30_H_40_N_5_O_7_	582.2922	582.2922	−0.08	Cyclodepsipeptides
14	JBIR 115	C_30_H_40_N_5_O_7_	582.2922	582.2922	−0.08	Cyclodepsipeptides

* Verruculogen was detected as a [M+H-H_2_O]^+^ adduct in this study.

## Data Availability

Not applicable.

## Supplementary Materials

The following supporting information can be downloaded at: https://www.mdpi.com/article/10.3390/jof8050514/s1, Figure S1: Neighbor-joining phylogenetic tree of HDACs; Figure S2: Relative expression of gene-encoding regulators of oxidative stress are different in the ∆*clr3* mutant when compared to wild type; Figure S3: UPLC-DAD chromatograms obtained for the crude extracts from (A) Δ*clr3* and (B) wild-type strains of *P. brasilianum*; Figure S4: HRESI-MS data for isoaustinone (1); Figure S5: HRESI-MS data for acetoxydehydroaustin (2); Figure S6: HRESI-MS data for Austinol (3); Figure S7: HRESI-MS data for Austinoneol (4); Figure S8: HRESI-MS data for brasiliamide A (5); Figure S9: HRESI-MS data for brasiliamide B (6); Figure S10: HRESI-MS data for brasiliamide C (7); Figure S11: HRESI-MS data for brasiliamide D (8); Figure S12: HRESI-MS data for brasiliamide E (9); Figure S13: HRESI-MS data for verruculogen (10); Figure S14: HRESI-MS data for verruculogen TR-2 (11); Figure S15: HRESI-MS data for penicillic acid (12); Figure S16: HRESI-MS data for JBIR 114 (13); Figure S17: HRESI-MS data for JBIR 115 (14); Figure S18: Mass spectrum of ion [M+H]^+^ *m*/*z* 439.1878 obtained for brasiliamide A (5) through DESI-IMS; Figure S19: Mass spectrum of ion [M+H]^+^ *m*/*z* 494.2287 obtained for verruculogen (10) through DESI-IMS; Table S1: Primers used in this study for construction of ∆*clr3* strain; Table S2: *Penicillium brasilianum* strains used in this study; Table S3: Individual genes selected for RT-qPCR analyses based on the orthology with oxidative stress regulation genes in *A. fumigatus*; Table S4: Primers used in this study for RT-qPCR analyses.
