# Supplementary material for: The Penicillium brasilianum Histone Deacetylase Clr3 Regulates Secondary Metabolite Production and Tolerance to Oxidative Stress
Source: J Fungi (Basel). 2022 May 17;8(5):514. doi: 10.3390/jof8050514 (PMC9146837; doi:10.3390/jof8050514)
Supplement: Supplementary file 1 [file jof-08-00514-s001.zip › jof-1727952-supplementary.pdf]

Supporting information for

The *Penicillium brasilianum* histone deacetylase *clr3* regulates secondary metabolite production and tolerance to oxidative stress.

Daniel Yuri Akiyama[†], Marina Campos Rocha[‡], Jonas Henrique Costa[†], Caroline Brandão Teles[§], Giuliana da Silva Zuccoli[§], Iran Malavazi[‡]* and Taicia Pacheco Fill[†]*

[†] Department of Organic Chemistry, Institute of Chemistry, State University of Campinas, Campinas, São Paulo, Brazil.

[‡] Department of Genetic and Evolution, Federal University of São Carlos, São Carlos, São Paulo, Brazil.

[§] Department of Biochemistry and Tissue Biology, Institute of Biology, State University of Campinas, Campinas, São Paulo, Brazil

*Corresponding author: taicia@unicamp.br (TF); imalavazi@ufscar.br (IM)

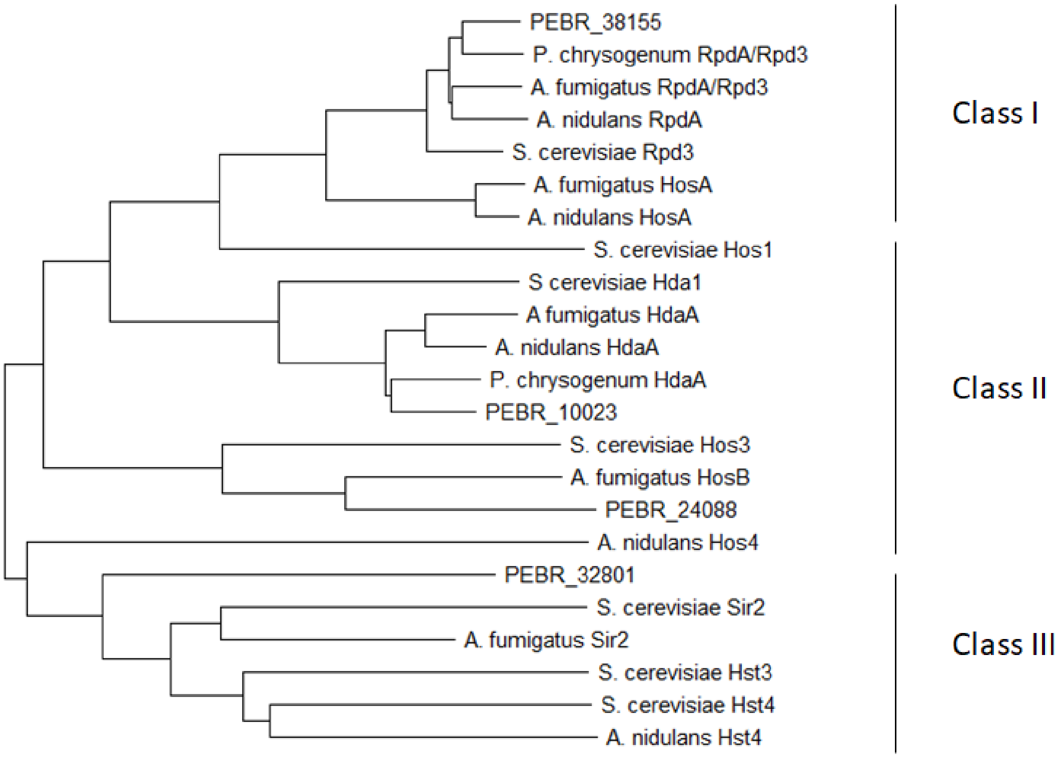

Figure S1. Neighbor-joining phylogenetic tree of HDACs. Histone deacetylase classifications are shown.

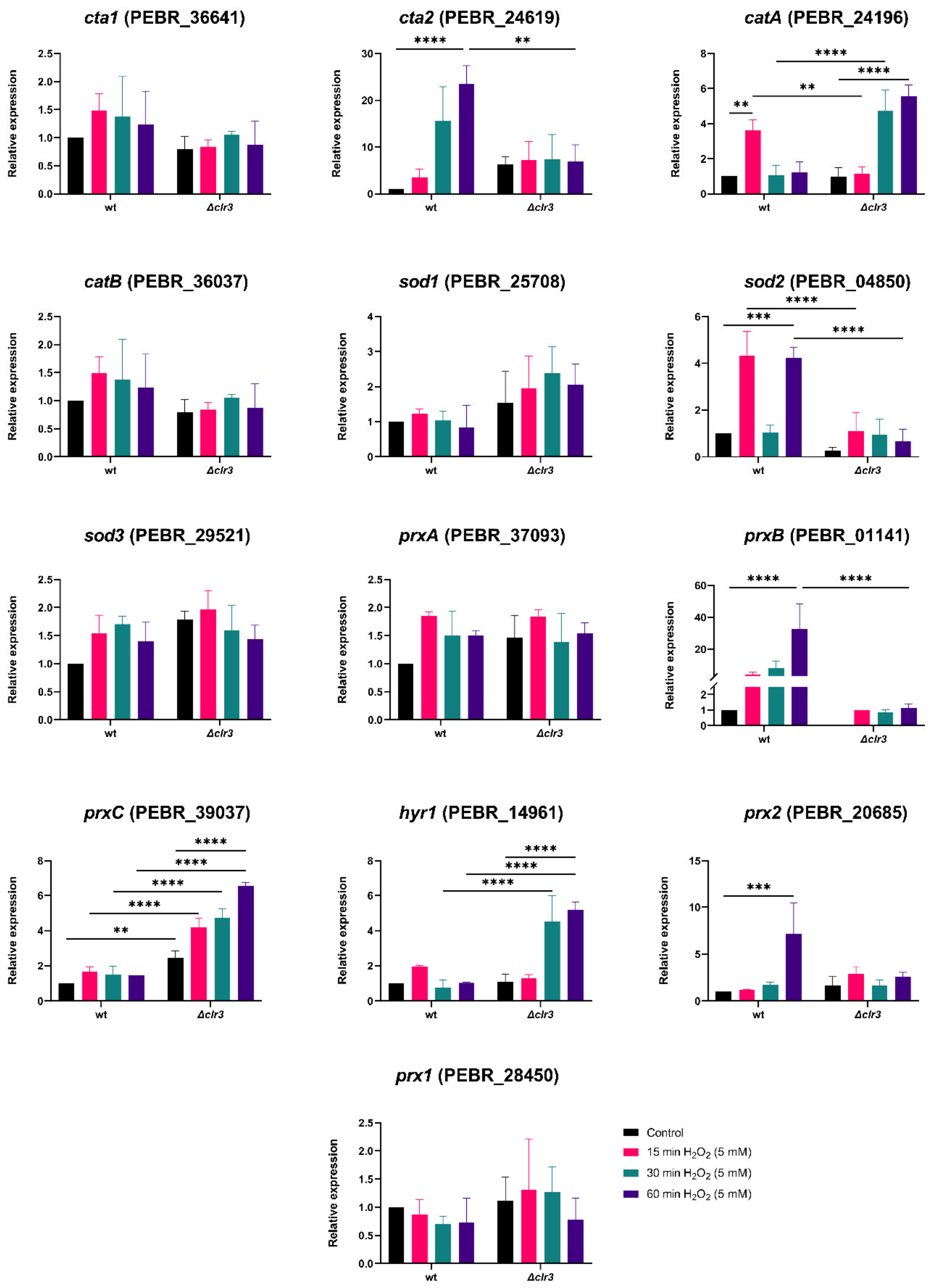

Figure S2. Relative expression of genes encoding regulators of oxidative stress are different in the Δ*clr3* mutant when compared to wild-type. The fold increase in each strain represents the normalized mRNA abundance relative to the wild-type strain. The data represent the average value of at least three independent experiments with two technical

repetitions each. The error bars represent standard deviation, **$p \leq 0.01$, ***$p \leq 0.005$, ****$p \leq 0.001$ (two-way ANOVA – Tukey's test, significance level 0.05).

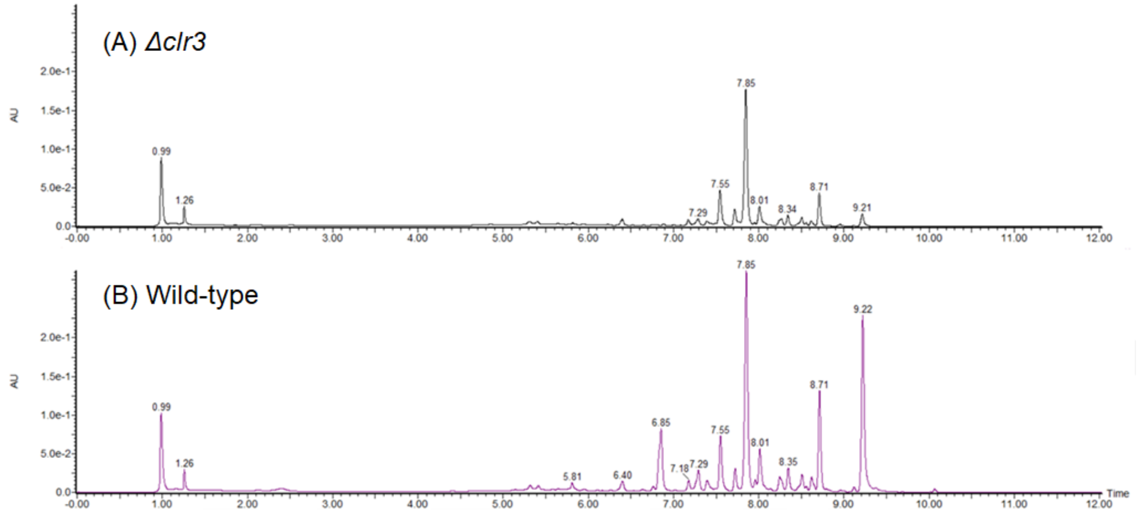

Figure S3. UPLC-DAD chromatograms obtained for the crude extracts from (A) Δ*clr3* and (B) wild-type strains of *P. brasilianum*. Chromatograms were plotted to the same scale.

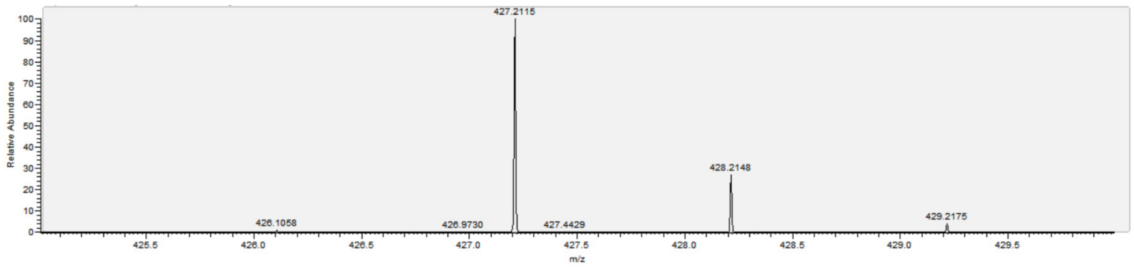

Figure S4. HRESI-MS data for isoaustinone.

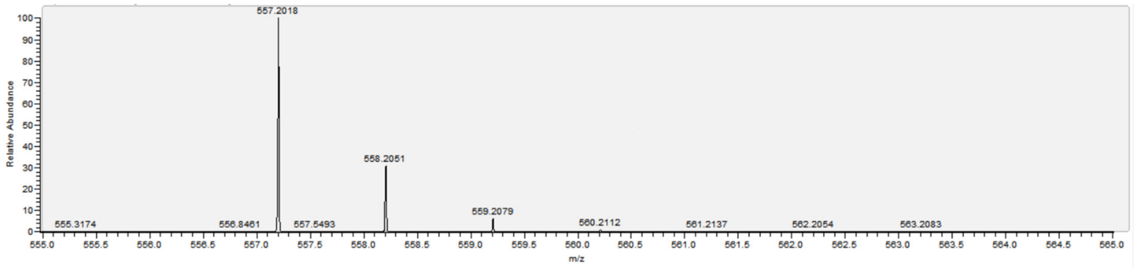

Figure S5. HRESI-MS data for acetoxydehydroaustin.

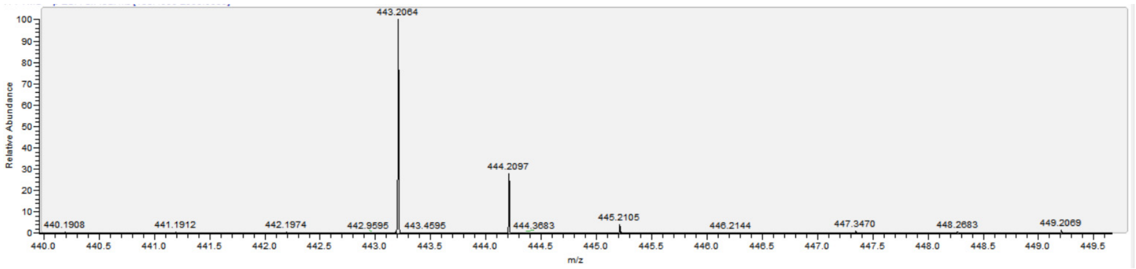

Figure S6. HRESI-MS data for Austinol.

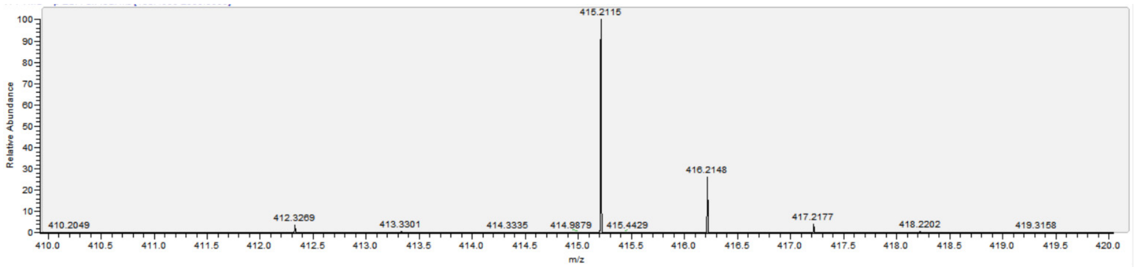

Figure S7. HRESI-MS data for Austinoneol.

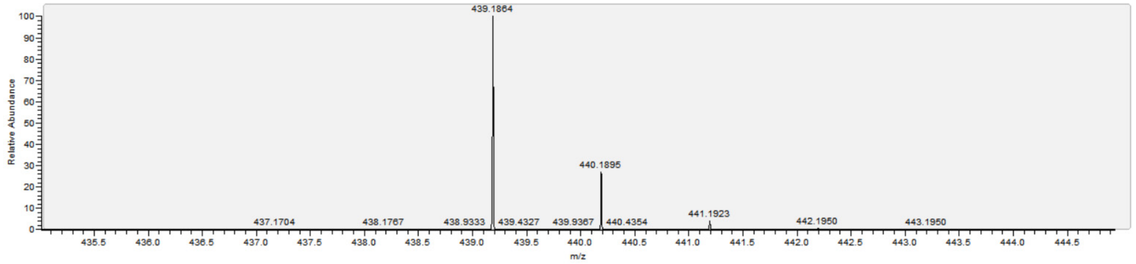

Figure S8. HRESI-MS data for brasiliamide A.

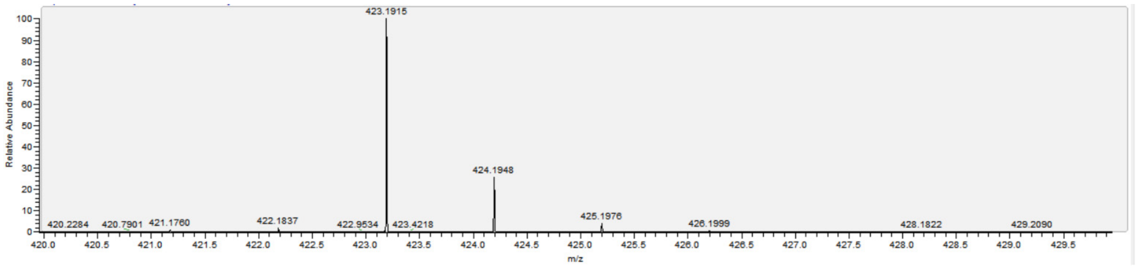

Figure S9. HRESI-MS data for brasiliamide B.

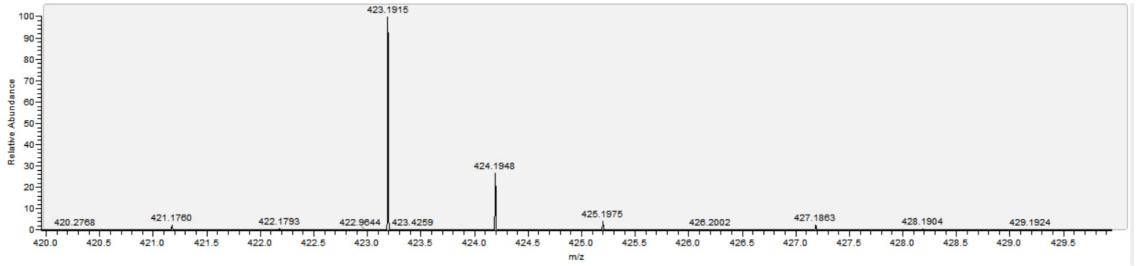

Figure S10. HRESI-MS data for brasiliamide C.

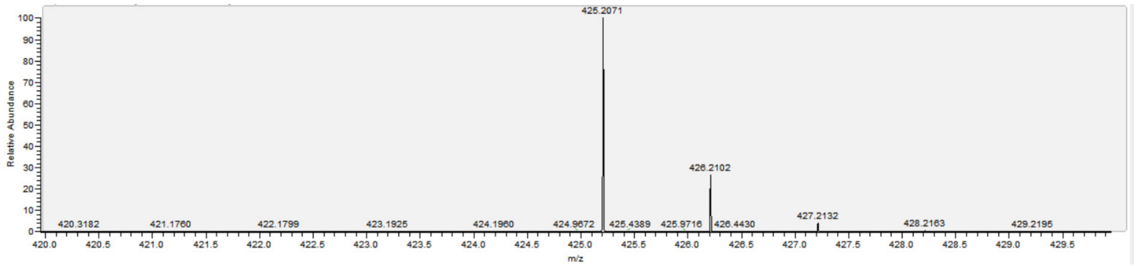

Figure S11. HRESI-MS data for brasiliamide D.

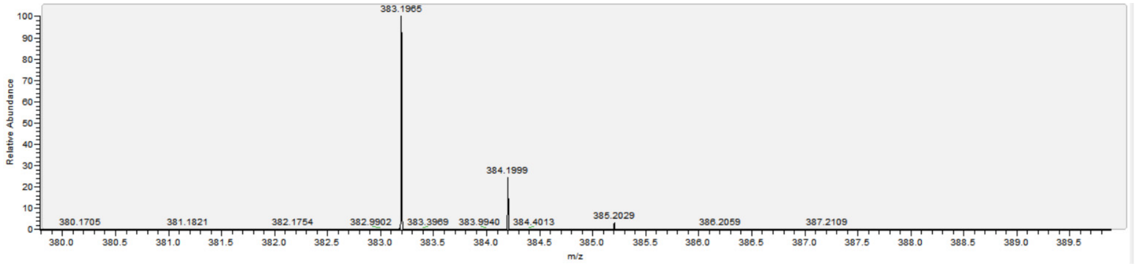

Figure S12. HRESI-MS data for brasiliamide E.

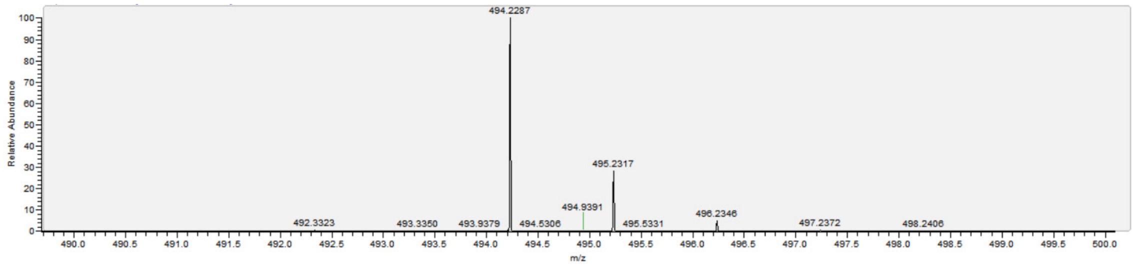

Figure S13. HRESI-MS data for verruculogen.

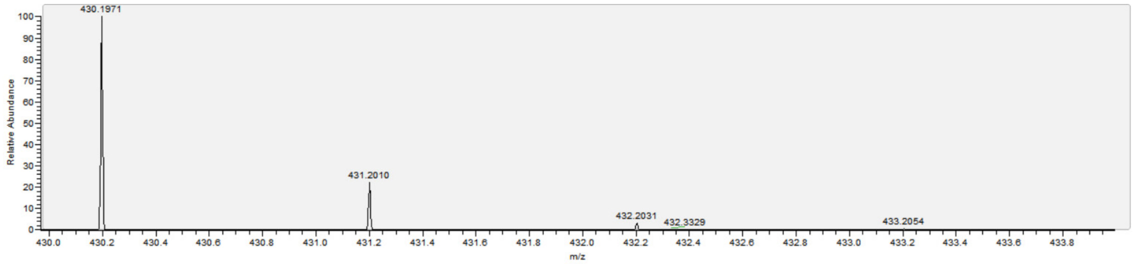

Figure S14. HRESI-MS data for verruculogen TR-2.

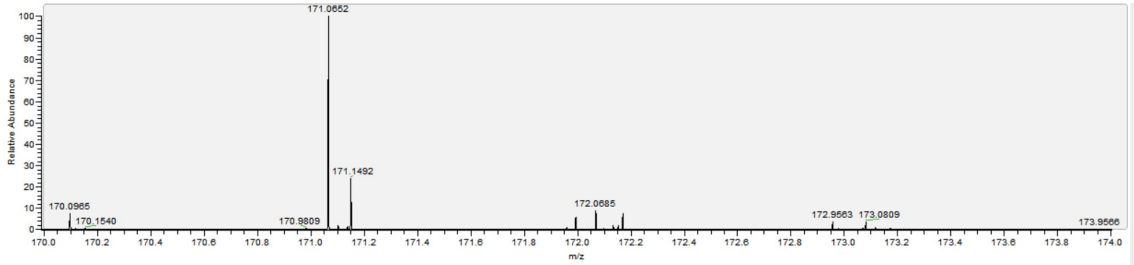

Figure S15. HRESI-MS data for penicillic acid.

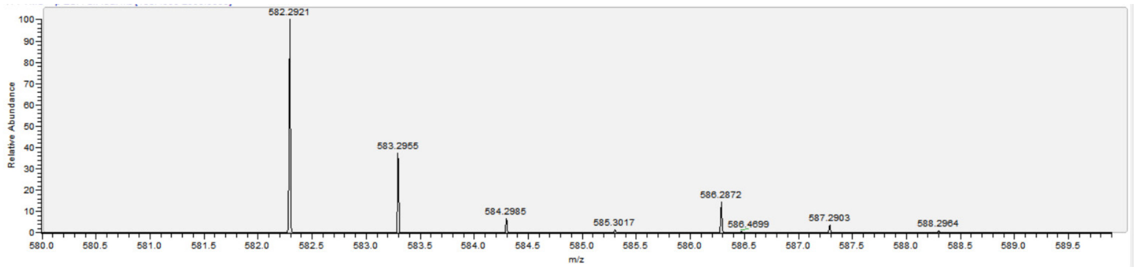

Figure S16. HRESI-MS data for JBIR 114.

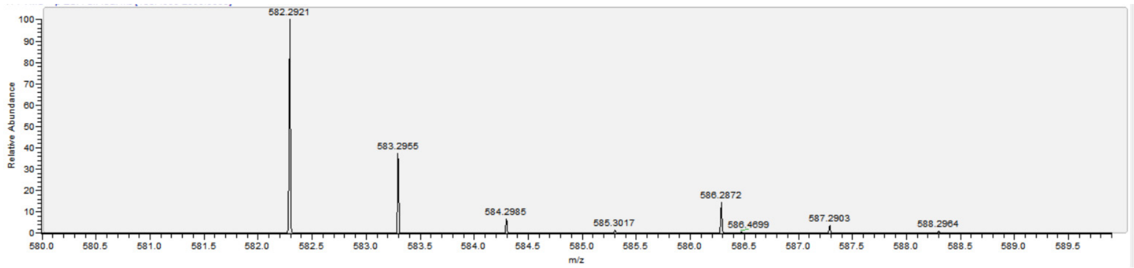

Figure S17. HRESI-MS data for JBIR 115.

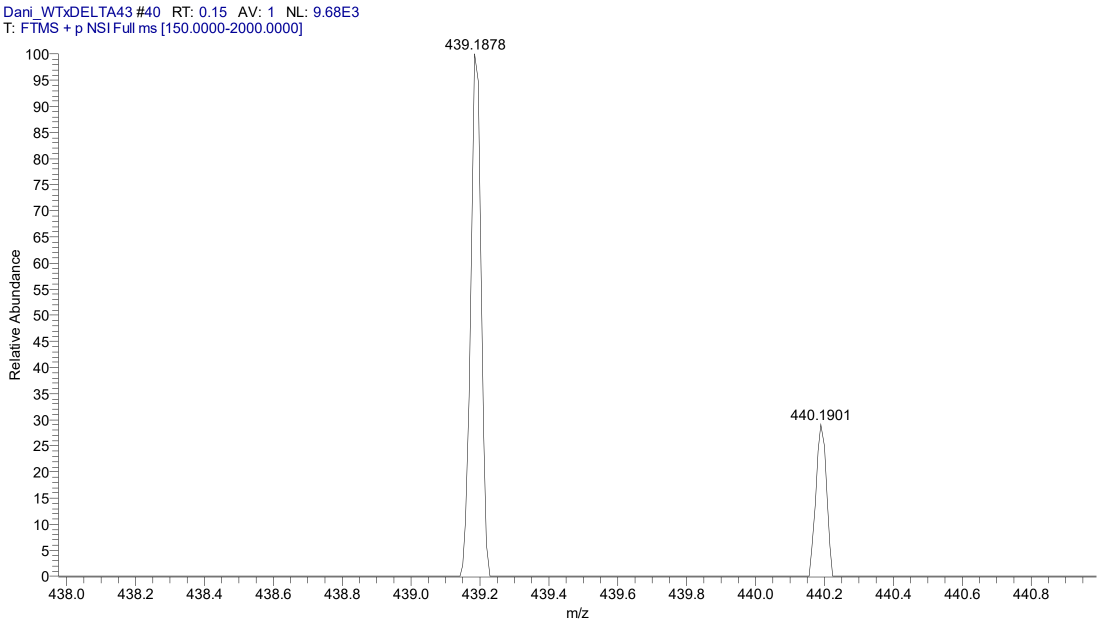

Figure S18. Mass spectrum of ion [M+H]+ *m/z* 439.1878 obtained for brasiliamide A through DESI-IMS.

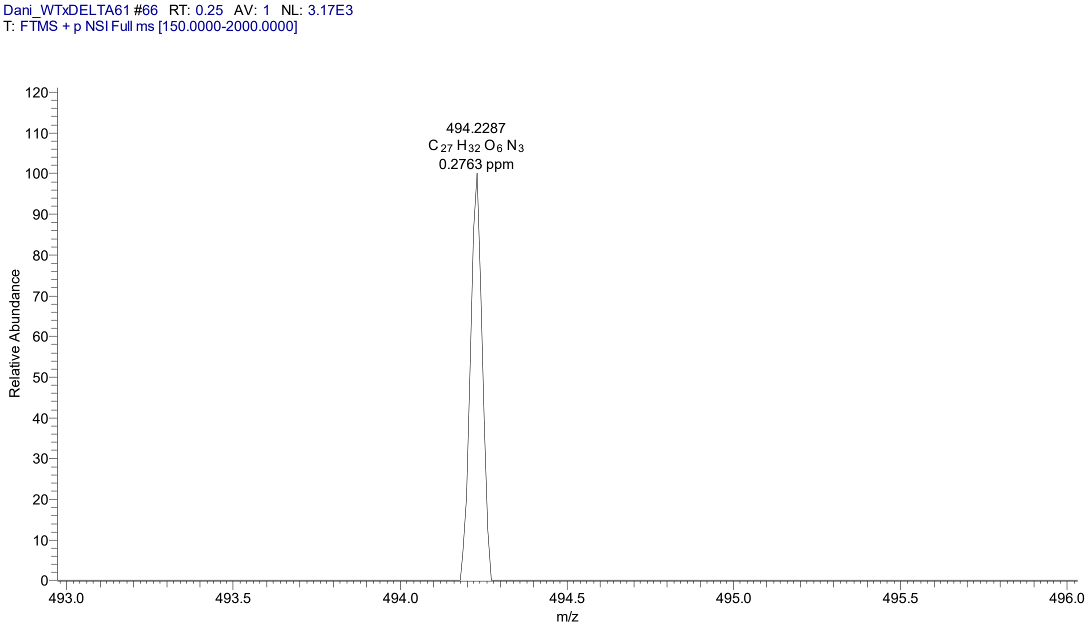

Figure S19. Mass spectrum of ion [M+H]+ *m/z* 494.2287 obtained for verruculogen through DESI-IMS.

Table S1. Primers used in this study for construction of Δ*clr3* strain.

Primer name	Sequence (5' → 3')
clr3 5F	GTAACGCCAGGGTTTTCCCAGTCACGACGAGCTTCGGTCGAAAGGTA
clr3 5R	tcagctaggtcaacgaataaggatgattctTTGCCAGGTGGAGAGAAG
hph F	AGAATCATCCTTATTCGTTGACCT
hph R	AGGTCGAGTGGAGATGTGGA
clr3 3F	aagcgcccactccacatctccactcgacctGTTGAAGCTAACGCCATC
clr3 3R	GCGGATAACAATTTCACACAGGAAACAGCCGAAGCAATAATCCATCG
clr3 500 ups	TCCGTCGTTGACCTGACTTG
clr3 F	CGTCAAGCGCGATAGTTTGG
clr3 R	GATCATCCACCAACCCAGCA

Small letters indicate homology to hygromycin gene fragment in the cassette.
Underlined letters indicate homology to the pRS426 flanking sequence.

Table S2. *Penicillium brasilianum* strains used in this study.

Name	Genotype	Reference
Wild-type	Wild-type (LaBioMMi 136)	(dos Santos Geris and Rodrigues-Fo 2002)
Δclr3	Δclr3::hph; Hyg^R	This study

Table S3. Individual genes selected for RT-qPCR analyses based on the orthology with oxidative stress regulation genes in *A. fumigatus*.

Gene	*A. Fumigatus* ortholog	Identity (%)	Similarity (%)	E-Value	DeepLoc prediction (likelihood)
prx1 (PEBR_28450)	AFUB_065670	83	90	7.10^{-133}	Cytoplasm (0.6905)
prx2 (PEBR_20685)	AFUB_080670	78	86	7.10^{-152}	Mitochondrion (0.993)
prxA (PEBR_37093)	AFUB_096050	85	90	8.10^{-102}	Cytoplasm (0.8825)
prxB (PEBR_01141)	AFUB_078510	80	90	2.10^{-104}	Mitochondrion (0.998)
prxC (PEBR_39037)	AFUB_049980	79	88	4.10^{-96}	Cytoplasm (0.9428)
hyr1 (PEBR_14961)	AFUB_036900	76	87	7.10^{-106}	Cytoplasm (0.6753)

sod1 (PEBR_25708)	AFUB_056780	87	97	3.10^{-98}	Mitochondrion (0.4173)
sod2 (PEBR_04850)	AFUB_014080	68	77	5.10^{-97}	Cytoplasm (0.6944)
sod3 (PEBR_29521)	AFUB_073150	82	90	0	Mitochondrion (0.9843)
cta1 (PEBR_36641)	AFUB_017280	43	58	6.10^{-128}	Peroxisome (0.7115)
cta2 (PEBR_24619)	AFUB_033720	44	57	4.10^{-133}	Peroxisome (0.7445)
catA (PEBR_24196)	AFUB_094400	64	77	0	Cytoplasm (0.5587)
catB (PEBR_36037)	AFUB_046060	51	65	2.10^{-153}	Extracelullar (0.8947)

Table S4. Primers used in this study for RT-qPCR analyses.

Gene	Primer name	Sequence (5' → 3')
prx1 (PEBR_28450)	PEBR_28450 F<br>PEBR_28450 R	ACCCCGAGCGCAAGGT<br>CGTTGGTGGTGTCCTGGTAGT
prx2 (PEBR_20685)	PEBR_20685 F<br>PEBR_20685 R	TGCCTTCCTGTACGACATGATT<br>TGCCCTTTTGGTCGATGTTAT
prxA (PEBR_37093)	PEBR_37093 F<br>PEBR_37093 R	CCGCTGACCGCTACAATCTC<br>GTGGTTGGCAGAGCAAACG
prxB (PEBR_01141)	PEBR_01141 F<br>PEBR_01141 R	AGGGCGACGCCATTCC<br>TTGTTGCCGGGAGAGTTCTC
prxC (PEBR_39037)	PEBR_39037 F<br>PEBR_39037 R	GGTCAGCTATGCGCAAATAGAGA<br>ACGGGATGCTCCTACCTTGA
hyr1 (PEBR_14961)	PEBR_14961 F<br>PEBR_14961 R	GCTTCCCCTGCAACCAGTT<br>GGACTGGATCTCATCGTTGGA
sod1 (PEBR_25708)	PEBR_25708 F<br>PEBR_25708 R	TGGCTCCAAGCAGGACAAG<br>CGCCAAGGACGCTCTCA
sod2 (PEBR_04850)	PEBR_04850 F<br>PEBR_04850 R	GCATCAATTGCCCCTCAAA<br>GGTTCAAGGGCCTGAAGGTAA
sod3 (PEBR_29521)	PEBR_29521 F<br>PEBR_29521 R	CACCGCATCCCCCAATT<br>TCCGGAACACCATTCTCCTT
cta1 (PEBR_36641)	PEBR_36641 F<br>PEBR_36641 R	CGCCACATGAACGGCTACT<br>GGTACCGTCCGGCTTGATC
cta2 (PEBR_24619)	PEBR_24619 F<br>PEBR_24619 R	TATCGCCTCGGCGTGAAC<br>GGACCTTGGCCGCGTTA
catA (PEBR_24196)	PEBR_24196 F<br>PEBR_24196 R	ACCAAGCGCAAACCTTCTACA<br>TTGGCAACGTGCAGCTTTT
catB (PEBR_36037)	PEBR_36037 F<br>PEBR_36037 R	CGCTGGATGATGCCCAGAT<br>GAACGTGAGTAGTCCTTCCTTC
clr3 (PEBR_10023)	PEBR_10023 F<br>PEBR_10023 R	CCACCTGATCGCCTCAAGAT<br>TGACGAGATTCCAGGGAAT
